# Tumor and germline next generation sequencing in high grade serous cancer: experience from a large population‐based testing program

**DOI:** 10.1002/1878-0261.12817

**Published:** 2020-10-22

**Authors:** Melanie Care, Jeanna McCuaig, Blaise Clarke, Sylvie Grenier, Raymond H Kim, Marjan Rouzbahman, Natalie Stickle, Marcus Bernardini, Tracy L. Stockley

**Affiliations:** ^1^ Laboratory Medicine Program Division of Clinical Laboratory Genetics University Health Network Toronto Canada; ^2^ Department of Molecular Genetics University of Toronto Toronto Canada; ^3^ Familial Cancer Clinic Princess Margaret Hospital Cancer Centre University Health Network Toronto Canada; ^4^ Lawrence S. Bloomberg Faculty of Nursing University of Toronto Toronto Canada; ^5^ Department of Laboratory Medicine and Pathobiology University of Toronto Toronto Canada; ^6^ Division of Medical Oncology Department of Medicine University of Toronto Toronto Canada; ^7^ Department of Gynecologic Oncology Princess Margaret Hospital Cancer Centre Toronto Canada

**Keywords:** BRCA1/BRCA2, germline variant, high‐grade serous cancer, next‐generation sequencing, somatic variant, tumor testing

## Abstract

The aim of this study was to determine the prevalence of somatic and germline pathogenic variants (PVs) in high‐grade serous cancer (HGSC) and to demonstrate the technical feasibility and effectiveness of a large‐scale, population‐based tumor testing program. It involved a retrospective review of genetic test results in 600 consecutive HGSC tumor samples and a subsequent comparison of germline and tumor results in a subset of 200 individuals. Tumor testing was successful in 95% of samples (570/600) with at least one *BRCA1/2* PV identified in 16% (93/570) of cases. Among the 200 paired cases, *BRCA1/2* PVs were detected in 38 tumors (19%); 58% were somatic (22/38); and 42% were germline (16/38). There was 100% concordance between germline and tumor test results. This is the largest series of *BRCA1/2* testing in HGSC (tumor‐only and paired cohorts), reported to date, and our data show that an effectively designed and validated population‐based tumor testing program can be used to determine both treatment eligibility and hereditary cancer risk.

## Graphical abstract

The presence of somatic or germline *BRCA1/2* pathogenic variants impacts the effectiveness of PARP inhibitor therapy in high‐grade serous ovarian cancer. This study demonstrates that a large‐scale tumor testing program can effectively and accurately identify both germline and somatic variants. Nearly 60% of pathogenic variants are somatic, emphasizing the importance of tumor testing in care pathways of this patient population.

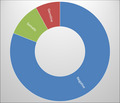

**Abbreviations**
CNVs: copy number variants
FFPE: formalin‐fixed paraffin‐embedded
HGSC: high‐grade serous cancer
NGS: next‐generation sequencing
PARPi: poly (ADP‐ribose) polymerase inhibitors
PV: pathogenic variant
VAF: variant allele fraction
VUS: variant of uncertain significance

## 1 Introduction

High‐grade serous cancers (HGSCs) of ovarian, fallopian tube, or primary peritoneal origin affect approximately 1 in 78 women over their lifetime and are the fifth leading cause of cancer deaths in women [1]. Large phase III clinical trials have demonstrated that treatment with poly (ADP‐ribose) polymerase inhibitors (PARPi) results in increased progression‐free and overall survival rates in women with HGSC associated with either inherited (germline) or acquired (somatic) *BRCA1 or BRCA2 (BRCA1/2)* pathogenic variants (PVs) [2, 3, 4, 5, 6]. Given these results, PARPi therapy is now recommended for women with platinum‐sensitive HGSC with either germline or somatic *BRCA1/2* PV [7, 8, 9].

Genetic testing of *BRCA1/2* has become the standard of care for all women with HGSC, with the primary goal of identifying hereditary cancer families to provide accurate risk assessment, surveillance, and risk reduction options for at‐risk individuals. [7, 10, 11] However, the introduction of PARPi in the treatment of HGSC is rapidly changing the landscape of *BRCA1/2* testing. While the prevalence of germline *BRCA1/2* PV is reported to be 10–20% in women with HGSC [12, 13], *BRCA1/2* PVs are identified in 15–30% of tumor samples [14, 15, 16, 17, 18, 19]. The difference, representing individuals with somatic variants present only in tumor tissue, accounts for an important cohort of patients who could benefit from PARPi therapy, but who would not be identified by germline testing alone.

Testing FFPE tumor tissue has the advantage of being able to identify somatic variants not detected through germline analysis. Tumor testing, when implemented as a reflex order by pathologists at the time of histologic diagnosis of HGSC, can also provide results to clinicians quickly while avoiding recognized barriers to germline genetic testing, including low referral rates for genetic counseling and restrictive testing guidelines [20, 21]. Consequently, *BRCA1/2* tumor testing is now recommended at the time of initial diagnosis of HGSC for the purposes of determining PARPi treatment eligibility [7, 8].

Despite these advantages, challenges exist in *BRCA1/2* tumor testing. Technical issues associated with using FFPE tissue for next‐generation sequencing (NGS) are an important consideration, including the potential for decreased success rates in samples obtained from biopsies versus resections, or those from patients who have undergone neoadjuvant chemotherapy due to potentially reduced sample size or quality. In addition, detection of large copy number variants (CNVs) is a recognized challenge and concerns have been raised around the potential for up to 5% of germline variants to be missed using tumor testing. [7, 22, 23]

It is also important to recognize that variants identified in tumor testing cannot be classified as being of either germline or somatic origin without testing of a paired germline sample [24]. Since the implications of carrying a germline *BRCA1/2* PV extend beyond cancer treatment to include risks for other cancers and to family members, individuals with positive tumor results still require appropriate genetic counseling and the option of germline testing. Further, the evaluation of *BRCA1/2* alone in tumor tissue will not identify pathogenic variants in other genes conferring hereditary ovarian cancer risk, which are found in 4–7% of women with ovarian cancer [17, 25].

In August 2018, the province of Ontario implemented clinical reflex tumor *BRCA1/2* testing as a funded, standard‐of‐care service for all cases of newly diagnosed HGSC and previously diagnosed cases with negative germline *BRCA1/2* testing. The University Health Network (UHN) Genome Diagnostics laboratory was one of two initially funded provincial laboratories, providing a unique opportunity to evaluate the detection rate of tumor *BRCA1/2* variants in a large, population‐based, tumor testing program. In conjunction with our Familial Cancer Clinic, which provides genetic counseling and clinical germline testing for HGSC patients, we were able to compare tumor and germline results to assess concordance of findings in *BRCA1/2* and other hereditary ovarian cancer genes, in the largest paired tumor germline cohort reported to date. The aim of this study was to use population‐based data to determine the prevalence of somatic and germline PV in HGSC and to demonstrate the feasibility and effectiveness of a large‐scale tumor testing program for the purposes of determining treatment eligibility and hereditary cancer risk.

## 2 Methods

### 2.1 Study population

A retrospective review was undertaken of all HGSC *BRCA1/2* tumor tests performed in the Genome Diagnostics Laboratory at University Health Network, in Toronto, between August 2018 and August 2019. This study conformed to the standards of the Declaration of Helsinki and was conducted under UHN Research Ethics Board oversight (#17‐5616).

Tumor testing was initiated in one of two ways: (a) reflexively by the reviewing pathologist following surgery or biopsy, at the time of histopathological diagnosis of HGSC; or (b) by the treating oncologist for patients who were platinum‐sensitive and had previously tested negative for a germline *BRCA1/2* pathogenic variant. Patients were recorded as being either an incident or prevalent case, with incident cases defined as those sent for *BRCA1/2* tumor testing within 6 months of procedure date (surgery or biopsy), and all others classified as prevalent cases. This distinction was made to assess for a potential bias within our cohort because individuals with a previously identified germline *BRCA1/2* PV were not eligible for tumor testing, potentially resulting in an under‐representation of germline variants in the prevalent group. Conversely, those with new diagnoses (incident cases) would be less likely to have undergone prior germline testing and are likely to provide a more accurate representation of the HGSC population.

Patients with available germline test results for review were identified through the clinical database of the Princess Margaret Cancer Centre Familial Cancer Clinic.

### 2.2 FFPE tissue samples and DNA extraction

All tumor samples were reviewed by a pathologist to confirm a diagnosis of HGSC. FFPE tissue samples (8 slides at 7‐μm thickness or two, 1‐mm cores) were macrodissected to enrich for tumor. Samples were digested overnight in Proteinase K (20 mg·mL^−1^), and DNA was extracted using a magnetic bead purification method designed for FFPE tissue (Maxwell 16 FFPE Plus LEV DNA Purification Kit; Promega, Madison, WI, USA) on an automated extractor (Maxwell 16; Promega). DNA concentration was evaluated using fluorometry (Qubit dsDNA Assay Kit on the Qubit 2.0 Fluorometer; Thermo Fisher Scientific, Waltham, MA, USA).

### 2.3 Tumor testing

A custom next‐generation sequencing (NGS) panel of 59 cancer predisposition genes (target region of 0.2 Mbs) including exons and intronic regions of *BRCA1* and *BRCA2* was used for tumor testing. Hybridization libraries (SureSelect XT; Agilent Technologies, Santa Clara, CA, USA) were sequenced on the Illumina platform (NextSeq 500; Illumina, San Diego, CA, USA), with a minimal read depth of 100× on the target regions. Data analysis used a custom bioinformatic analysis program, with reads aligned to the reference human genome (hg19) using the Burrows‐Wheeler Aligner (bwa‐mem) [26]. Duplicate reads were marked using Picard Mark Duplicates (Broad Institute, Cambridge, MA, USA) followed by application of the Genome Analysis Toolkit (gatk v3.3‐0) best practices recommendations (Broad Institute) for Base Quality Score Recalibration (bqsr) algorithm. Variant calling used varscan v2.3.8, [27] and copy number used CNVkit v0.9.3. [28] For CNV analysis, a panel of normal (PoN) created by CopywriteR was used, with a target bin size of 100 bp (rather than default of 267 bp) to obtain a higher‐resolution segmentation for on‐target sequencing coverage depth of 200× to 300×. For any CNVs identified by NGS testing as within a possible deletion (log2 ratio less than −0.8) or duplication (log2 ratio greater than 0.8) range, verification was performed using the multiple ligation probe amplification kits P002, P087, and P090 (MRC‐Holland, Amsterdam, the Netherlands).

### 2.4 Tumor variant classification

Identified variants were filtered to remove technical artifacts and benign changes and retain only potentially clinically relevant variants (Alissa Clinical Informatics Platform, Agilent). Tumor variants were evaluated by the 2015 ACMG germline variant interpretation guidelines [29]. All variants meeting criteria for pathogenic or likely pathogenic (P/LP) classification were reported and for the purposes of the study, reported as PV. Variants of uncertain significance (VUS) were also identified and reported as such.

### 2.5 Germline testing

Germline testing was performed as a clinical service through one of several non‐UHN accredited laboratories, on genomic DNA extracted from a peripheral blood sample. Germline testing included either *BRCA1*/*2*‐only testing, or a multigene cancer panel (including *BRCA1*/*2*), depending on the request of the clinical team. All germline variants were independently reviewed and classified by the study team according to the 2015 ACMG Variant Interpretation Guidelines [29].

### 2.6 Data collection and analysis

Data were collected through a retrospective review of pathology reports, clinical records, and genetic test results. Relevant data included procedure type (surgery vs biopsy), age at procedure date, primary tumor site, incident versus prevalent cases, neoadjuvant chemotherapy status, and tumor *BRCA1/2* results [including specific variant information, variant classification, and variant allele fraction (VAF)].

For cases in which germline test results were available, the type of testing (*BRCA1/2* only vs multigene panel testing), identified gene variants, and family history information were recorded. In the subset of cases for which germline results were available, tumor and germline results were compared to determine the proportion of somatic versus germline variants, and concordance of findings between tumor and germline testing.

Descriptive statistics were used to summarize participant characteristics. In the full tumor cohort, significant differences in the frequency of PV among prevalent and incident cases of HGSC were assessed using the Fisher exact test or Pearson chi‐square test, as appropriate. One‐way ANOVA was used to compare differences in age at procedure among four possible groups of tumor results (*BRCA1* PV, *BRCA2* PV, negative, and VUS); pairwise differences were reported using the Bonferroni post hoc test. In the paired tumor and germline cohort, the Fisher exact test or Pearson chi‐square test was used, as appropriate, to identify significant differences in the frequency of PV, as well as the proportion of somatic versus germline variants, among prevalent and incident cases. Statistical analyses were completed using ibm spss statistics for Windows, version 24 (IBM Corp, Armonk, NY, USA); statistical significance was reported using a two‐tailed α = 0.05.

## 3 Results

### 3.1 Study subjects

A total of 603 samples, from 600 patients, were received for *BRCA1/2* tumor testing at UHN within the study period, including 518 surgical resections (2 from distant sites), 81 biopsies, 2 cytology samples, and 2 with unknown origin. Of the 603 samples, 3 (0.5%) were duplicate samples, 2 (0.3%) had insufficient tumor FFPE material to attempt extraction, and 28 (4.6%) had sufficient tumor DNA isolated to attempt testing but gave inconclusive technical results on NGS, for a total of 570 individuals for whom tumor results were available. Of these, 45% (256/570) were incident, 54% (307/570) were prevalent, and 1% (7/570) were considered other types of cases (metastases, recurrences, information not provided). All were HGSCs, with ovarian primaries being the most prevalent site of origin (240/570; 42%). The remainder were fallopian tube (111/570; 20%), tubo‐ovarian (99/570; 17%), primary peritoneal (43/570; 7.5%), or undeterminable (77/570; 13.5%) primary sites. The mean age at the time of procedure (surgery or biopsy) was 63.1 years (range: 33–90) (Table 1).

**Table 1:** Demographics and overall *BRCA1/2* tumor results of study population. PV, pathogenic variant; VUS, variant of uncertain significance; NEG, no reported variants; other, includes metastases, recurrence, unknown/information not provided; undetermined, primary tumor site could not be determined.

	ALL (*n* = 570)	*BRCA1*‐PV (*n* = 63)	*BRCA1*‐VUS (*n* = 18)	*BRCA2*‐PV (*n* = 30)	*BRCA2*‐VUS (*n* = 33)	NEG (*n* = 426)
Age at procedure^a^	63.1	56.8^b^	64.4	61.9	63.5	64.1
Mean (years); range	33–90	33–83	51–79	38–84	43–84	38–90
Incident/prevalent						
Incident	256 (45%)	27 (43%)	10 (56%)	12 (40%)	13 (39%)	194 (45.5%)
Prevalent	307 (54%)	35 (55.5%)	8 (44%)	18 (60%)	20 (61%)	226 (53%)
Other	7 (1%)	1 (1.5%)	0	0	0	6 (1.5%)
Primary tumor site						
Ovarian	240 (42%)	31 (49%)	6 (33%)	14 (47%)	17 (52%)	158 (37%)
Fallopian tube	111 (19.5%)	12 (19%)	5 (28%)	5 (16.5%)	5 (15%)	84 (20%)
Tubo‐ovarian	99 (17.5%)	8 (13%)	4 (22%)	5 (16.5%)	5 (15%)	75 (17.5%)
Primary peritoneal	43 (7.5%)	6 (9.5%)	0	5 (16.5%)	3 (9%)	32 (7.5%)
Undetermined	77 (13.5%)	6 (9.5%)	3 (17%)	1 (3.5%)	3 (9%)	70 (18%)

^a^ Age at time of procedure was used as not all samples were collected at the time of diagnosis.

^b^ Significantly different from the patients with mutation‐negative tumors (*P* < 0.001).

### 3.2 Tumor testing

Test success rates were slightly lower in biopsy samples versus surgical resection, with 90% (73/81) of biopsy samples yielding NGS results versus 96% (497/518) for resections. Of the 60 tumor samples collected following neoadjuvant chemotherapy, 59 were successfully tested and only 1 (1.7%) resulted in an inconclusive result due to a high number of failed regions on NGS.

**Fig. 1:**
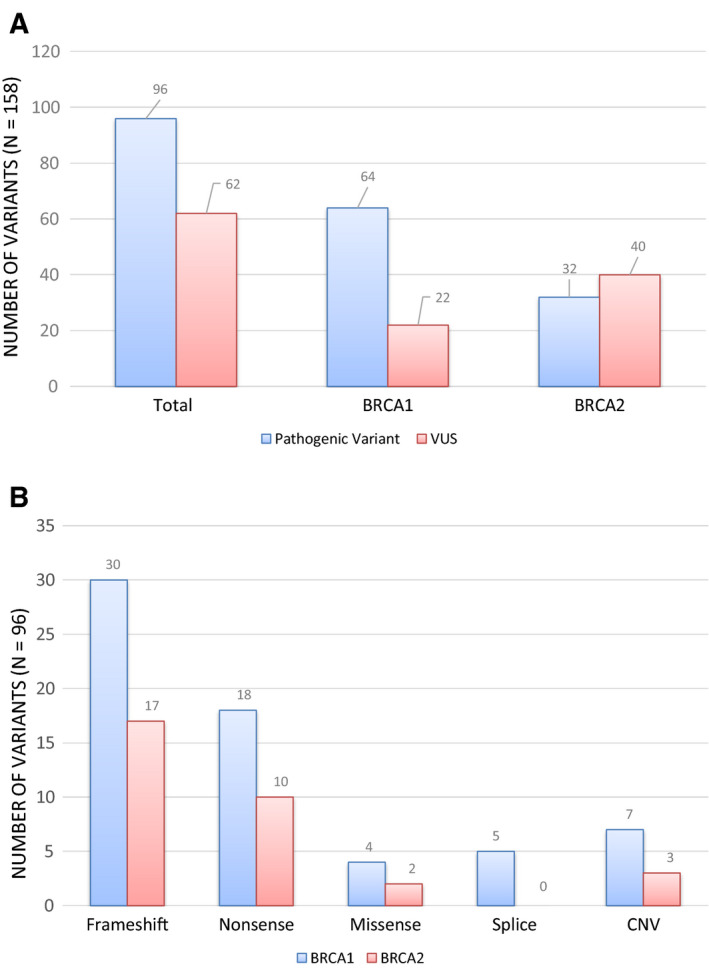
*BRCA1* and *BRCA2* variants in tumor samples (*n* = 570): (A) A total of 158 *BRCA1/2* variants were identified: 96 pathogenic variants (PVs) and 62 variants of uncertain significance (VUS); (B) types of pathogenic variants identified in tumor samples, stratified by gene (*BRCA1* or *BRCA2*).

A total of 96 *BRCA1/2* PVs were identified in 93 samples, for an overall detection rate of 16% (93/570) (Fig. 1A). The detection rate of *BRCA1/2* PV was not significantly different in incident (15%; 39/256) versus prevalent (17%; 53/307) cases (*P* = 0.517). Single *BRCA1* PVs were the most frequent, identified in 10.9% of samples (62/570), and single *BRCA2* PVs were identified in 5% (28/570). Two PVs were identified in 0.5% (3/570) of tumors. In addition, 62 VUS [*BRCA1* (22); *BRCA2* (40)] were identified in 60 tumors, either in isolation or together with another *BRCA1/2* VUS or PV.

A significant difference was noted in the mean age at time of procedure among women with and without a *BRCA1* PV identified in their tumor tissue. The mean age of women with *BRCA1* PV was significantly younger than those with negative (56.81 years vs 64.05; *P* < 0.001) or VUS results (56.81 years vs 63.80; *P* = 0.001); the difference between women with *BRCA1* and *BRCA2* PVs was not significant (56.81 years vs 61.87 years; *P* = 0.143). Differences between negative, VUS, and *BRCA2* groups were not statistically significant.

**Fig. 2:**
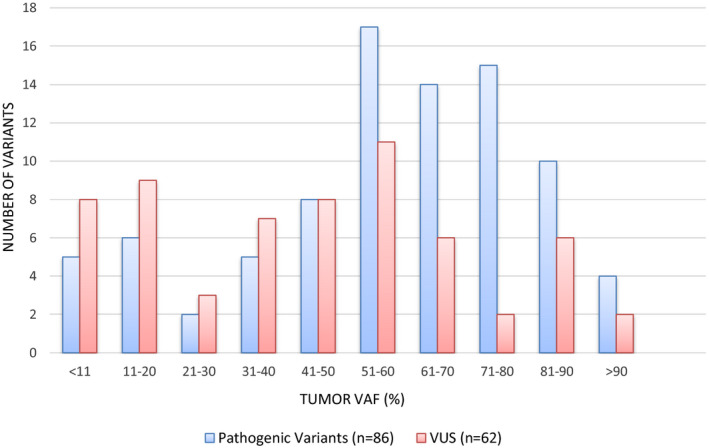
Variant allele fraction (VAF) of *BRCA1/2* tumor variants. Comparison of VAF of PV versus VUS identified on tumor testing. A higher proportion of PVs were present at a VAF > 40% as compared to VUS (76% vs 56%).

The majority of identified *BRCA1/2* PVs were frameshift (47/96; 50%) or nonsense (28/96; 29%) variants (Fig. 1B). Missense and splicing variants account for 6% (6/96) and 5% (5/96), respectively. Ten large CNVs were identified (Table S1). The VAF of *BRCA1/2* PV ranged from 8% to 97% (Fig. 2). Excluding large CNVs, 76% (65/86) of PV had a VAF greater than 40% on the tumor test. The VAF of VUS ranged from 5% to 95%; however, only 56% (35/62) were present at a VAF higher than 40%.

### 3.3 Tumor and germline paired analysis

**Fig. 3:**
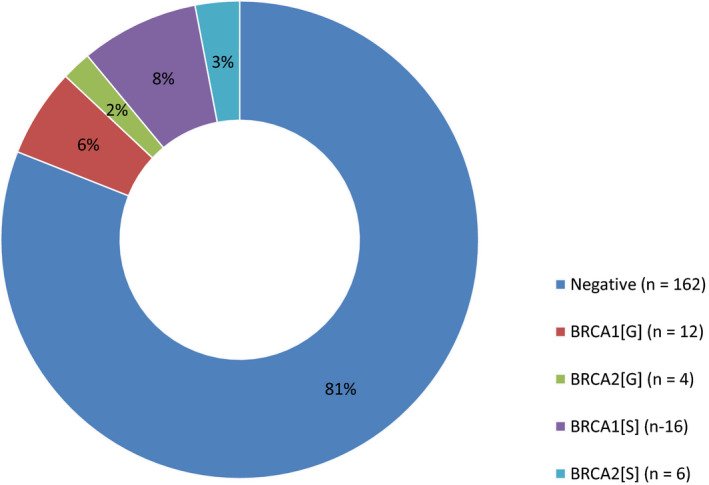
Origin of *BRCA1/2* pathogenic variants identified in paired cohort (*n* = 200). Results demonstrating the *BRCA1/2* PV profile of paired tumor and germline samples. Negative—refers to samples carrying no variants or VUS; [G]—confirmed germline origin; [S]—confirmed somatic origin.

Within the paired cohort, 19% (38/200) of tumors had *BRCA1/2* PV; 74% (28/38) were in *BRCA1;* and 26% (10/38) were in *BRCA2*. A direct comparison between tumor and germline results showed that 42% (16/38) of the tumor PV were present in the germline and the remaining 58% (22/38) were absent, thus confirming a somatic origin (Fig. 3). All 37 germline *BRCA1/2* variants (PV and VUS) were identified on tumor testing, demonstrating 100% detection of germline variants through FFPE tumor testing.

**Fig. 4:**
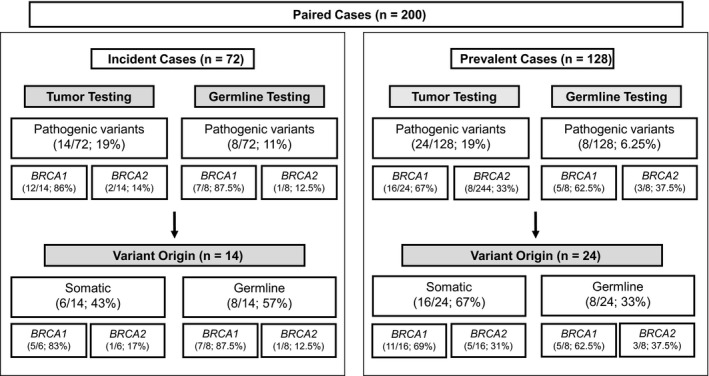
Variants detected in a paired tumor germline cohort. Direct comparison of paired tumor and blood analyses in incident versus prevalent cases. All variants detected through germline testing were identified in tumor samples. To determine somatic versus germline origin, the number of variants identified by germline testing is subtracted from the number of variants identified in tumors.

The paired cohort consisted of 72 incident and 128 prevalent cases. The overall frequency of *BRCA1/2* PV in tumor samples was 19% (38/200), and there was no difference between the incident (14/72) and prevalent (24/128) groups (*P* = 0.904). Of the identified PV, 58% (22/38) were somatic and 42% (16/38) were germline. A greater proportion of somatic PVs were identified in the prevalent cases (67%; 16/24) as compared to incident cases (43%; 6/14); however, this difference was not statistically significant (*P* = 0.152) (Fig. 4).

**Fig. 5:**
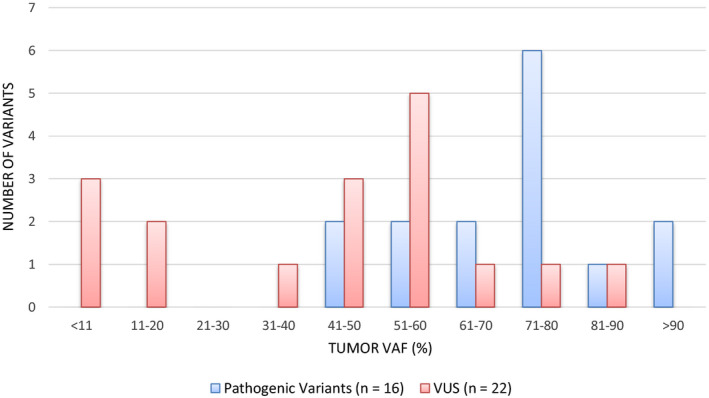
Tumor variant allele fraction (AF) of confirmed germline variants. Distribution of observed VAF in 32 confirmed germline sequence variants. Six germline variants, all VUS, were identified in tumors at VAF < 40%, whereas all germline PVs were present in tumors at VAF > 40%.

When comparing somatic *BRCA1/2* PV (*n* = 22) with germline *BRCA1/2* (*n* = 16), somatic PVs were more likely to be nonsense (27% vs 19%) or copy number variants (27% vs 6%) and less often frameshift variants (36% vs 62.5%). With the exception of the large CNVs, the *BRCA1/2* VAF in tumor samples for confirmed germline variants (PV and VUS) ranged from 5% to 94% (Fig. 5). However, the tumor VAF of germline *BRCA1/2* PV (*n* = 15) was all greater than 44% VAF (44–94%), versus the germline VUS (*n* = 17) which ranged from 5% to 90% VAF.

Of those individuals whose germline testing included multigene panels, 6% (10/163) had LP/P variants identified in genes other than *BRCA1/2 (BRIP1*–3; *MLH1*–1; *RAD51C*–3; *RAD51D*–3). When the NGS data from these tumor samples were reanalyzed to include additional genes on the panel, all 10 variants were found in the tumor data (Table S2). Review of family history information in these 10 cases demonstrated that only one individual had a family history of ovarian cancer. However, all but one of these individuals had a reported family history of HBOC‐related cancers in third degree or closer relatives.

## 4 Discussion

With the introduction of PARPi therapy, the identification of both germline and somatic *BRCA1/2* PVs is a critical part of the treatment pathway for HGSC [7, 8, 9]. As a consequence, there is a new urgency for tumor *BRCA1/2* testing in HGSC to ensure timely identification of all patients eligible for PARPi therapy, including those who may be missed through traditional service delivery models aimed at germline variant detection.

The results of our study show that large‐scale tumor testing is both effective and feasible. In our cohort, there was a relatively low test failure rate of 4.6%, the majority of which represented samples from one referral center suggesting a site‐specific issue with fixation methods or storage of FFPE material. Importantly, we reported a high success rate in biopsy tissue and in patients who had neoadjuvant chemotherapy. Both are important considerations for tumor testing in HGSC, as many patients are not candidates for primary surgery given the aggressive nature of the disease. Our results demonstrate the ability of tumor testing in multiple clinical scenarios, irrespective of a patient's primary treatment plan.

We have also demonstrated that NGS, accompanied by appropriate bioinformatic assessment, allows for the identification of large CNVs on FFPE tissue. CNV detection is a recognized challenge due to tumor heterogeneity, DNA fragmentation, and nucleic acid modifications that occur in the fixation process of tumor tissue [30], but is paramount to the success of a tumor testing program. In this study, 10% (10/96) of *BRCA1/2* PV identified in tumor samples were large CNVs (Table S1). Seven of these were somatic, representing a significant proportion of PARPi‐eligible patients that would not have been identified by germline testing alone and further emphasizing the importance of CNV detection using tumor testing methodologies.

The overall prevalence of *BRCA1/2* PV in 570 HGSC tumor samples was 16%. While consistent with the 17% reported in a recently published large cohort, [14] it is somewhat lower than the 24–30% reported by others [15, 18, 31, 32]. This is potentially a reflection of the large‐scale, population‐based testing approach represented in our study compared with small, controlled research‐based settings. For example, sample submissions from multiple sites with pathology reviewed by multiple pathologists may introduce additional variables that are difficult to control for, but may be more reflective of real‐world scenarios.

Evaluation of the paired tumor germline cohort in this study provides important insights into *BRCA1/2* variants in HGSC, which, in turn, has implications for service delivery planning and genetic testing models. The proportions of somatic versus germline variants have varied widely, with 14–45% of tumor PV reported to be of somatic origin [2, 14, 15, 31, 32]. In our paired cohort, the largest published to date, 58% of *BRCA1/2* PVs were somatic. Though not statistically significant, a smaller proportion of somatic PVs were identified in incident versus prevalent cases (43% vs 67%). This was expected, as tumor testing was not completed for prevalent cases with known germline *BRCA1/2* PV. Therefore, the proportion of somatic PVs is likely overrepresented in the prevalent group and 43% may be more accurate estimate of somatic variants in HGSC. This suggests that almost half of PARPi‐eligible individuals may be missed through germline *BRCA1/2* testing alone, potentially resulting in a failure to initiate appropriate therapy. In addition, the identification of germline *BRCA1/2* variants in 33% of the prevalent cases demonstrates that individuals with germline PV may be missed by existing germline‐focused care models.

Comparing tumor and blood results also provides interesting insights into the variant allele fraction (VAF) of confirmed germline variants. While all germline *BRCA1/2* PVs were identified in tumor at VAFs over 40% (VAF = 44–94%), confirmed germline VUS were seen at VAF in tumors as low as 5% and as high as 90%. Although VAF may appear to be a convenient predictor of variant origin, there are currently no established cutoff values of tumor VAF for confirmed germline variants [33]. Our data demonstrate the potential pitfalls associated with using VAF to determine variant origin and highlight the necessity of confirmatory germline testing in order to provide appropriate genetic counseling and risk assessment.

The feasibility, benefits, and limitations of a tumor‐first testing model have been recently discussed, given its potential to reduce turnaround times and overall test volumes by negating the need for follow‐up germline testing in individuals with negative tumor results [34]. The technical ability to identify all variants through tumor testing is of utmost importance when considering the potential of a tumor‐first testing model, particularly given the challenges associated with testing on FFPE tissue, and reports that up to 5% of germline variants will be missed with tumor analysis [7, 34]. In our paired cohort of 200 patients, we demonstrated a 100% detection of 37 germline *BRCA1/2* variants (PV and VUS) with tumor testing, including one large copy number variant. As described, the ability to detect CNVs in FFPE tissue is exceptionally important in this context, given that they are reported to account for 10% of germline *BRCA1/2* variants [35, 36]. While current guidelines recommend germline *BRCA1/2* testing for all women diagnosed with epithelial ovarian cancer [7], our data suggest that with appropriate validation and high technical standards, a tumor‐first testing model may be a viable option to detect both somatic and germline variants.

In addition to technical considerations, ethical concerns with a tumor‐first model have also been raised. Given the potential for opportunistic germline variant identification through tumor testing, there is a question of informed consent and an individual's right to decline knowledge of hereditary cancer risk [37, 38]. The proportion of somatic variants detected in our study suggests that tumor testing in more in keeping with the definition of screening, as opposed to diagnostic testing, for hereditary cancer. Consequently, oncologists speaking to individuals about tumor *BRCA1/2* testing can do so in the context of therapeutic implications, with more detailed informed consent discussions around hereditary testing occurring at the time of genetic counseling following a positive tumor test.

Though the benefit of *BRCA1/2* tumor testing is increasingly evident for the purposes of therapeutic decision‐making, the importance of identifying non‐*BRCA1/2*‐associated hereditary cancer families cannot be overlooked. Germline variants in other ovarian cancer risk genes are identified in 4–7% of ovarian cancer [17, 25], and *BRCA1/2*‐only tumor testing will result in a failure to identify and manage these individuals at increased cancer risk. We have demonstrated that multigene panel testing on FFPE tumor samples can be used to detect germline variants beyond *BRCA1/2*, as 10 germline variants in other ovarian cancer risk genes were identified in tumor testing (*BRIP1, MSH2, RAD51C, RAD51D*) (Table S2). Importantly, in these cases family history alone may not have been sufficient to trigger a referral to a hereditary cancer clinic for assessment. Thus, prior to eliminating secondary germline testing for HGSC patients with negative tumor results, tumor‐first workflows should include multigene panels to avoid missing hereditary cancer families. Such a model could reduce lengthy wait times often experienced in hereditary cancer clinics by allowing them to focus on germline confirmation of positive tumor results and cascade testing [39, 40].

## 5 Conclusions

The data presented here demonstrate that a large, population‐based tumor testing program in HGSC is effective in identifying *BRCA1/2* PV both for the purposes of determining treatment eligibility, and as a potential screen for hereditary cancer syndromes. As tumor testing for patients with HGSC becomes increasingly available across healthcare organizations, collaborative efforts between oncology, pathology, molecular diagnostics, and genetic teams will help to determine best practices and care pathways to maximize the benefits for patient care.

## Conflict of interest

Melanie Care has received speaker honoraria and travel support from AstraZeneca, Inc. Jeanna McCuaig has speaker honoraria and travel support from AstraZeneca, Inc., and speaker honoraria from Pfizer, Inc. Tracy L. Stockley has received funding for test development from AstraZeneca and honoraria for advisory board meetings. All other authors declare no conflicts of interest.

## Author contributions

MC conceived and designed the study; acquired, analyzed, and interpreted the data; and wrote the original draft and revisions. JM conceived and designed the study; acquired, analyzed, and interpreted the data; and wrote the original draft and revisions. BC involved in study concept; acquired data; wrote the manuscript; and critically reviewed and revised the manuscript. SG involved in study concept; acquired data; wrote the manuscript; and critically reviewed and revised the manuscript. RHK involved in study concept; acquired data; wrote the manuscript; and critically reviewed and revised the manuscript. MR involved in study concept; acquired data; wrote the manuscript; and critically reviewed and revised the manuscript. NS involved in study concept; acquired data; wrote the manuscript; and critically reviewed and revised the manuscript. MB involved in study concept; acquired data; wrote the manuscript; and critically reviewed and revised the manuscript. TLS involvedconcevied and designed the study; acquired, analyzed and interpreted the data; and wrote the original draft and revisions.

## Supporting information


**Table S1.** Large copy number variants (CNVs) identified in tumor samples.
**Table S2.** Non‐BRCA1/2 pathogenic and likely pathogenic variants identified through multigene panel testing.
