# Corrigendum to: Tumor and germline next generation sequencing in high grade serous cancer: experience from a large population‐based testing program

**DOI:** 10.1002/1878-0261.13018

**Published:** 2021-05-31

The authors of [1] would like to correct the name of the author Marcus Bernardini to Marcus Q Bernardini.
