# Supplementary material for: Tumor and germline next generation sequencing in high grade serous cancer: experience from a large population‐based testing program
Source: Mol Oncol. 2020 Oct 22;15(1):80–90. doi: 10.1002/1878-0261.12817 (PMC7782089; doi:10.1002/1878-0261.12817)
Supplement: Supplementary file 1 — Table S1. Large copy number variants (CNVs) identified in tumor samples. Table S2. Non‐BRCA1/2 pathogenic and likely pathogenic variants identified through multigene panel testing. [file MOL2-15-80-s001.docx]

**Supplementary Table 1: Large copy number variants (CNV) identified in tumor samples**

| **Gene** | **Copy Number Variant** | **Origin (Germline vs Somatic)** |
| --- | --- | --- |
| *BRCA1* | Exon 3 duplication | Somatic |
| *BRCA1* | Exon 4-6 duplication | Somatic |
| *BRCA1* | Exon 7 deletion | Germline |
| *BRCA1* | Exon 8 deletion | Somatic |
| *BRCA1* | Exon 22-23 deletion | Not available |
| *BRCA2* | Exon 25-27 deletion | Somatic |
| *BRCA1* | Whole gene deletion | Not available |
| *BRCA1* | Whole gene duplication | Somatic |
| *BRCA2* | Exon 21-24 deletion | Somatic |
| *BRCA2* | Whole gene deletion | Somatic |

**Supplementary Table 2. Non-*BRCA1/2* pathogenic and likely pathogenic variants identified through multi-gene panel testing**

| **Gene** | **Variant** | **Reported Tumor VAF (%)** | **ACMG Class** | **Primary HGSC Site** | **Age at Procedure (years)** | **Personal Hx other cancer  (type; age)** | **Reported Family Hx – OVCA** | **Reported Family Hx – Other HBOC** |
| --- | --- | --- | --- | --- | --- | --- | --- | --- |
| *BRIP1* | p.Ala402Valfs*21 | 70 | P | PP | 53 | N | N | Y |
| *BRIP1* | p.Ser624* | 84 | P | OVB | 68 | N | N | Y |
| *BRIP1* | p.Trp816* | 91 | P | TOB | 51 | N | N | Y |
| *MLH1^* | p.Arg487* | 51 | P | FTU | 51 | Endometrial; 51 | N | Y |
| *RAD51C* | p.Thr132Asnfs*23 | 65 | P | FTU | 57 | Breast; 49 | N | Y |
| *RAD51C* | p.Ser234* | 64 | P | OVB | 68 | Breast; 51 | Y | Y |
| *RAD51C* | c.904+5G>T | 26 | LP | UK | 70 | N | N | N |
| *RAD51D* | p.Gln151* | 86 | P | OVB | 50 | N | N | Y |
| *RAD51D* | p.Arg253* | 69 | P | OVU | 54 | Thyroid; 46 | N | Y |
| *RAD51D* | p.Trp268* | 94 | P | OVB | 56 | N | N | Y |

All variants were initially identified in germline multi-gene panel testing, with subsequent confirmation of presence in tumor samples. Personal cancer history and reported family histories are provided. VAF – variant allele fraction; P – pathogenic; LP –likely pathogenic; OVU – unilateral ovary; OVB – bilateral ovary; FTU – unilateral fallopian tube; PP – primary peritoneal; TOB – bilateral tubo-ovarian; UK – unknown; OVCA – ovarian cancer; ^ - previously identified variant (prior to multi-gene cancer panel testing); Other HBOC – other cancers within the hereditary breast/ovarian cancer spectrum; includes breast cancer, melanoma, pancreatic cancer or prostate cancer
